# Proanthocyanidins Protect Epithelial Cells from Zearalenone-Induced Apoptosis via Inhibition of Endoplasmic Reticulum Stress-Induced Apoptosis Pathways in Mouse Small Intestines

**DOI:** 10.3390/molecules23071508

**Published:** 2018-06-21

**Authors:** Miao Long, Xinliang Chen, Nan Wang, Mingyang Wang, Jiawen Pan, Jingjing Tong, Peng Li, Shuhua Yang, Jianbin He

**Affiliations:** Key Laboratory of Zoonosis of Liaoning Province, College of Animal Science & Veterinary Medicine, Shenyang Agricultural University, Shenyang 110866, China; longjlau@126.com (M.L.); 20152529@stu.syau.edu.cn (X.C.); LCwangnan@163.com (N.W.); m13940546231@163.com (M.W.); panjiawen0101@163.com (J.P.); m18641260406_1@163.com (J.T.)

**Keywords:** proanthocyanidins, zearalenone, oxidative damage, endoplasmic reticulum stress, intestinal epithelial cells, mice, apoptosis

## Abstract

This study evaluated the protective effect of proanthocyanidins (PCs) on reducing apoptosis in the mouse intestinal epithelial cell model MODE-K exposed to zearalenone (ZEA) through inhibition of the endoplasmic reticulum stress (ERS)-induced apoptosis pathway. Our results showed that PCs could reduce the rate of apoptosis in MODE-K cells exposed to ZEA (*p* < 0.01). PCs significantly increased the ZEA-induced antioxidant protective effects on the enzymes superoxide dismutase (SOD) and glutathione peroxidase (GSH-Px) and on the content of GSH. PCs also significantly decreased the ZEA-induced increase in the content of malondialdehyde (MDA). The analysis indicated that ZEA increased both mRNA and protein expression levels of C/EBP homologous protein (CHOP), GRP78, c-Jun N-terminal kinase (JNK), and cysteinyl aspartate specific proteinase 12 (caspase-12) (*p* < 0.05), which are related to the ERS-induced apoptosis pathway. ZEA decreased levels of the pro-apoptotic related protein Bcl-2 (*p* < 0.05) and increased the anti-apoptotic related protein Bax (*p* < 0.05). Co-treatment with PCs was also shown to significantly reverse the expression levels of these proteins in MODE-K cells. The results demonstrated that PCs could protect MODE-K cells from oxidative stress and apoptosis induced by ZEA. The underlying mechanism may be that PCs can alleviate apoptosis in mouse intestinal epithelial cells by inhibition of the ERS-induced apoptosis pathway.

## 1. Introduction

The gastrointestinal tract (GIT) is the primary tissue site of interactions with food contaminants [1]. Various toxic substances such as mycotoxins, which often exist in contaminated food or feeds, can damage intestinal epithelial cells [2]. Amongst the mycotoxins, zearalenone (ZEA), produced by many *Fusarium* species [3], is considered a common contaminant in food and feedstuffs [4]. ZEA has been implicated in reproductive disorders, as it can bind and activate estrogenic receptors [5]. ZEA has also shown multiple toxicities in the immune system [6], liver [7], and kidney [8]. In addition, it has carcinogenic potential [9] and enhances lipid peroxidation [10], which are most likely a result of its oxidative stress properties [11,12].

Recent studies have shown that ZEA can alter intestinal villous structures [13], affect the intestinal epithelial integrity of porcine cells [14], induce significant changes in the gene expression of porcine intestinal cells [15], and reduce the expression of junction proteins of intestinal cells [16]. As ZEA can damage the intestine, strategies to alleviate its harmful effects on the GIT represent an area of increasing interest.

Oxidative stress can induce cellular damage and dysfunction. Endoplasmic reticulum stress (ERS) is also intimately connected with oxidative stress. Some studies have shown that antioxidants can reduce levels of ERS [17,18]. It has also been shown that ZEA exerts its cytotoxic effects by causing both oxidative stress and ERS [19,20,21], suggesting that antioxidants could be used to prevent or attenuate stresses induced by ZEA. Studies have provided evidence demonstrating that some natural antioxidants can prevent almost all ZEA toxicities. The studies concluded that when mice were given crocin (250 mg/kg·b.w.), this could protect against ZEA-induced toxicity in cardiac tissue [22]. Studies have also shown that lycopene can inhibit inflammation and reproductive damage induced by ZEA when male Swiss albino mice received lycopene (20 mg/kg·b.w.) for 10 days [23]. Meanwhile, isothiocyanate from the Tunisian radish can also prevent genotoxicity induced by ZEA both in vivo and in vitro [24]. Aqueous extracts (250 μg/mL) could protect against ZEN-induced DNA damage in Vero cells [25]. Furthermore, studies have demonstrated that dietary vitamin C (150 mg/kg) can prevent ZEN-induced reproductive toxicity as well as immune and hematological toxicities in piglets [26,27]. Quercetin could reduce ERS and apoptosis induced by α- and β-zearalenol in HCT116 cells [28].

Proanthocyanidins (PCs) are the most effective natural antioxidants capable of scavenging free radicals in the body [29]. Previous studies have shown that PCs, as a result of antioxidant activity, prevented damage of the granulosa cells induced by 2.5 mg/mL D-gal when cells were co-treated with PCs at 5 μg/mL for 72 h [30]. In diabetic rats, a diet containing 250 mg/kg PCs was shown to protect against skeletal muscle damage by alleviating oxidative stress and ERS [31]. PCs have also been shown to decrease the bladder damage in diabetic rats when given orally at a dose of 250 mg/kg for 8 weeks [32]. PCs have also been shown to alleviate acute inflammation induced by LPS in rats when pre-treated with 200 mg/kg·d.w. for 15 days [33]. Other reports have also shown attenuation of cisplatin- and cadmium-induced testicular damage by inhibiting the oxidative/nitrative stress in rat testes for rats that were given 100, 200, or 400 mg/kg·d.w. doses [34,35,36]. PCs also prevented renal injury induced by amikacin and DOCA-salt hypertension in rats [37,38], attenuated lead-induced liver oxidative damage in Kunming mice by oral co-administration at 100 mg/kg for 6 weeks [39], and prevented steroid-induced osteonecrosis in rabbits given 100 mg/kg·b.w. for 14 consecutive days [40].

These studies have demonstrated that PCs can inhibit oxidative stress and apoptosis induced by many exogenous compounds. Our previous studies have shown that PCs protect against ZEA-induced testicular oxidative damage and Sertoli cell apoptosis via the Nrf2/ARE signaling pathway [41,42]. However, it is not clear whether PCs alleviate ZEA-induced intestinal cell apoptosis via inhibition of ERS-induced apoptotic pathways. In this study, the main purpose was to investigate whether PCs could protect against apoptosis in mouse intestinal epithelial cells, MODE-K, via inhibition of ERS-induced apoptosis pathways. This study provides further supporting evidence that PCs can alleviate the toxic effects of ZEA.

## 2. Experimental Section

### 2.1. Materials

ZEA (Sigma, St. Louis, MO, USA) was dissolved in diethyl sulfoxide. The stock solution of ZEA was 200 mg/mL and was stored at −20 °C. PCs were extracted from grape seeds with a purity of at least 95% (Hefei BoMei Science and Technology Co., Ltd., Hefei, China); these contained oligomeric proanthocyanidins (88.36%), catechin (6.68%), and l-epicatechin (4.54%). Kits were used for the testing of glutathione peroxidase (GSH-Px), total superoxide dismutase (T-SOD), glutathione (GSH), lactate dehydrogenase (LDH), and malondialdehyde (MDA) (Nanjing Jiancheng Bioengineering Institute, Nanjing, China). The cell counting Kit-8 (CCK-8) was used for the determination of cell viability in cell proliferation and cytotoxicity assays (Beijing TransGen Biotech Co., Ltd., Beijing, China). The following were used: 2-(4-amidinophenyl)-6-indolecarbamidine dihydrochloride (DAPI dihydrochloride) (Sigma Aldrich, St. Louis, MO, USA); the SYBR green real-time PCR (RT-PCR) kit (Takara, Otsu, Japan); the RevertAid First Strand cDNA Synthesis Kit (MBI Fermentas, Burlington, ON, Canada); the BCA Protein Assay Kit for detecting protein concentration (Thermo Fisher Scientific, Waltham, MA, USA); the preservation solution of RNA samples and the kits for total cell RNA extraction (Beijing Solarbio Science & Technology Co., Ltd., Beijing, China). The primers for cysteinyl aspartate specific proteinase 12 (caspase-12), C/EBP homologous protein (CHOP), c-Jun N-terminal kinase (JNK), the 78 kDa glucose-regulated protein (GRP78/Bip), and β-actin were synthesized by Meiji Bio Medical Science and Technology Co., Ltd. (Shanghai, China); anti-GRP78/Bip, -CHOP, -p-CHOP, -JNK, -p-JNK, -caspase-12, -Bax, and -β-actin monoclonal antibodies were used (Cell Signaling Technology, Boston, MA, USA). The antibodies were conjugated with secondary goat anti-mouse and goat anti-rabbit horseradish peroxidase (HRP) (Beijing TransGen Biotech Co., Ltd., Beijing, China). The small intestinal epithelial cell line MODE-K was used (Shanghai GuanDao Biological Engineering Co., Ltd., Shanghai, China).

### 2.2. Effect of ZEA and PCs on Cell Viability

The MODE-K cells were counted using a cell counting plate. The concentration was adjusted to be 1 × 10^5^ cells/mL; these were then were inoculated into 96-well plates. The number of cells per well was 1 × 10^4^. After inoculation, the cells were placed in a 37 °C, 5% CO_2_ incubator and cultured for 24 h. After the cells had adhered, 90 μL of serum-free medium and 10 μL of ZEA were added. The concentrations of ZEA were determined to be 0 (blank control), 10, 20, 40, 60, 80, 100, and 120 μmol/L.

The concentrations of PCs were set to 0, 5, 10, 20, 30, 40, 60, 80, and 100 μg/mL, respectively. In each treatment group, for four replicates, the cells and ZEA were co-cultured for 24 h; then 10 μL of CCK8 solution was added to a 96-well plate. After CCK-8 was added, the drug-treated cells were further cultured for 3 h, and the absorbance (OD value) of the cell solution was measured at 450 nm with a microplate reader (Infinite 200 PRO, ABI, Waltham, MA, USA). The survival rate of the intestinal epithelial cells was calculated by measuring the OD value of each group. The relative survival rate = (OD value of each treatment group/OD value of blank control group) × 100%. The half inhibition concentration (IC_50_) of the cells was calculated on the basis of the relative survival rate of the cells in each test-treatment group. The IC_50_ values were calculated by using SPSS19.0 software (IBM, Almon, NY, USA).

### 2.3. Effect of Different Concentrations of PCs Co-Treated with ZEA on Cell Viability

The tests were grouped into a control group, a ZEA group (65 μmol/L), PC groups (5, 10, and 15 μg/mL), and co-treated groups (ZEA concentration: 65 μmol/L; PC concentrations: 5, 10, and 15 μg/mL), with four replicates in each group, and the cells were harvested after 24 h of culturing. The supernatant was strictly analyzed by a colorimetric assay for detecting the content of nicotinamide adenine dinucleotide (NADH) at 340 nm in accordance with the operating procedures of the LDH kit.

### 2.4. Detection of Oxidation Indexes in Intestinal Epithelial Cells

The test groups were consistent with those mentioned above. The supernatants of the cells were used to measure the various oxidation indexes of the cells. The content of MDA and GSH and the enzyme levels of T-SOD and GSH-Px were detected by using assay kits for the oxidation indexes.

### 2.5. Cell Apoptosis Assay

The test groups were consistent with those mentioned above. The cells were digested with trypsin without eathylene diamine tetraacetic acid (EDTA) after being cultured for 24 h; then the cells were added to complete medium to stop digestion. The cell suspension was collected and transferred to a centrifuge tube. The supernatant of the cells was discarded after centrifugation. Then the impurities from the cells were washed away after the cells were suspended and mixed. When the cell surface residual substances were completely washed away after repeating three times, the cells were suspended and mixed by adding 100 μL of binding buffer. The cell suspension was gently mixed and then placed at room temperature for 15 min after 5 μL of fluoresceine isothiocyanate (FITC) and 10 μL of propidium iodide (PI) dye were added. For the additional three blank control groups, one group had only FITC added, one group had only PI added, and one group had only the binding buffer added; after 15 min, to each blank control group was added 400 μL of binding buffer. Finally, all the cells were filtered by using a 300-mesh cell strainer, and the filtered cell suspension was transferred to a flow tube. The apoptosis of the cells was detected by flow cytometry.

### 2.6. Real-Time PCR Analysis

The total RNA of the MODE-K cells was extracted by using Trizol. We used agarose gel electrophoresis to test whether the RNA was degraded or not. The RNA concentration and purity were detected using a Nucleic acid spectrometer (BU730, Beckman Coulter, Brea, CA, USA). The OD_260_/OD_280_ value should be between 1.8 and 2.0. The reverse transcription of RNA was performed using a reverse transcription kit. The primers were designed using Primer 5.0 software (IBM, Almon, NY, USA), and the specificity of the primers was test using Oligo 7 (IBM, Almon, NY, USA), before being synthesized. The primer sequences we used were as shown in Table 1. RT-PCR was performed by using an ABI Fluorescence Quantitative PCR instrument (iQ5, ABI, Waltham, MA, USA). The system configuration and operation were performed on ice according to Takara’s PrimeScript RT Reagent RR047A Kit. The 20 μL reaction system included 0.8 μL of forward primer and reverse primer, 2 μL of cDNA, 6.4 μL of dH_2_O, and 10 μL of SYBR Premix Ex Taq. A two-step reaction was used to set the cycler settings. The first step was predenaturation at 95 °C for 30 s; the second step was 40 repeated cycles for the PCR reaction at 95 °C for 5 s and at 60 °C for 34 s. The RT-PCR data were analyzed by the method of gene expression (i.e., 2^−ΔΔ*C*t^).

### 2.7. Western Blot Analysis

About 150 μL of RIPA cell lysate was added to each well of collected cells; then 1.5 μL of phenylmethylsulfonyl fluoride (PMSF) protein inhibitor was added, shaken vigorously for 15 s on ice, and incubated for 30 min. After the mixture was centrifugated at 14,000× *g* for 10 min in a 4 °C centrifuge, the supernatant was the protein solution. The BCA protein quantitative method was used to detect the sample protein concentration. SDS–polyacrylamide gel electrophoresis was used to separate the total protein. Afterwards, the isolated protein was transferred to a nitrocellulose membrane and was then blocked using 5% BSA for 2 h. Then, the primary antibodies of β-actin, JNK, p-JNK, GRP78/Bip, caspase-12, CHOP, and p-CHOP were incubated at 4 °C for 12 h in the blocking solution. Afterwards, the biotinylated anti-goat IgG antibody was added to the membranes and incubated for 2 h at room temperature after they were washed with TBST three times. Then, SA-HRP was used to incubate the membranes for 30 min at room temperature. After washing, DAB staining was used to visualize the immunoreactive bands at room temperature for 5 min. Finally, an image-analysis system was used to analyze the density of these target proteins.

### 2.8. Statistical Analysis

SPSS 19.0 (IBM, Almon, NY, USA) was used to analyze the data between groups, and multiple comparisons were performed using the LSD method. The test results were expressed as means ± standard deviations; *p* < 0.01 and *p* < 0.05 indicated that the differences between the experimental groups were extremely significant and significant, respectively.

## 3. Results

### 3.1. The Effect of ZEA and PCs on the Relative Survival of MODE-K Cells

**Figure 1:**
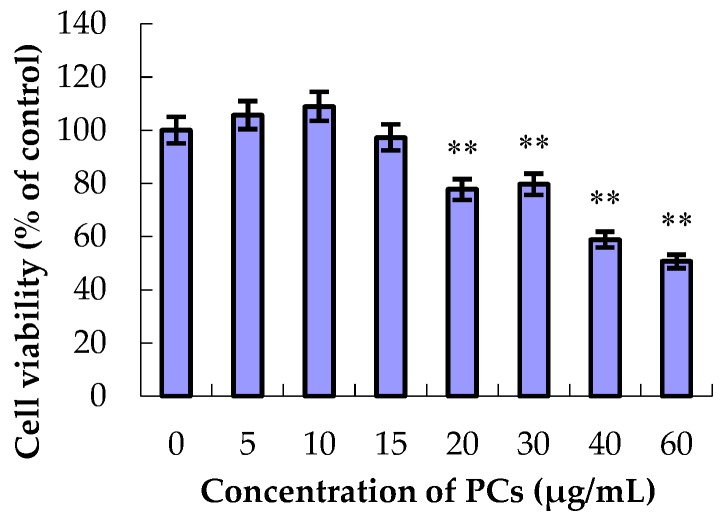
Effects of different concentrations of proanthocyanidins (PCs) on the viability of MODE-K cells. The relative survival rates of MODE-K cells did not decrease at PC concentrations of 5, 10, or 15 μg/mL. However, when the concentration of PCs was 20 μg/mL or higher, the relative survival rate of the MODE-K cells decreased significantly. ** *p* < 0.01 vs. control group.

As shown in Figure 1, the different concentrations of PCs had differential effects on the relative survival rate of MODE-K cells. Compared with the control group, the relative survival rates of MODE-K cells showed no decrease at PC concentrations of 5, 10, and 15 μg/mL. However, when the concentration of PCs was 20 μg/mL or higher, the relative survival rate of the epithelial cells decreased significantly (*p* < 0.01). Thus, in this study, 5, 10, and 15 μg/mL of PCs were added to the MODE-K cells.

**Figure 2:**
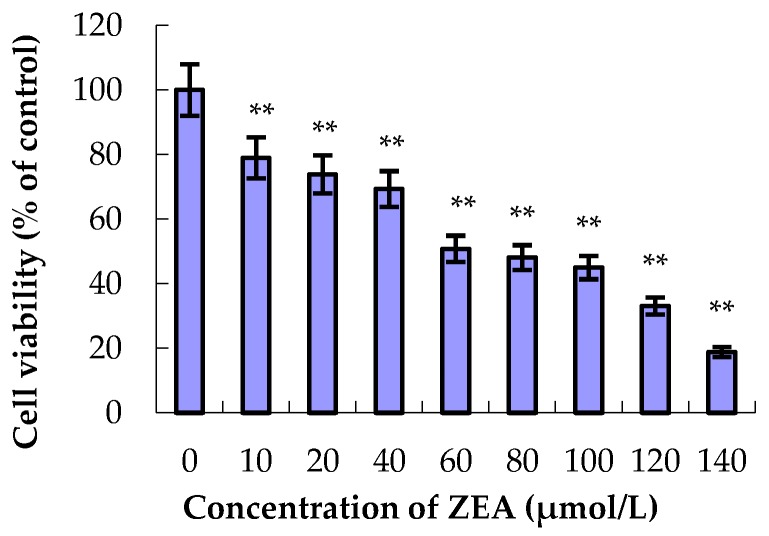
Effects of zearalenone (ZEA) on the viability of MODE-K cells. The relative survival rate of MODE-K cells gradually decreased with the increase in ZEA concentration when the cells were treated with ZEA for 24 h. The half-inhibitory concentration of ZEA on MODE-K cells was 65 μmol/L. ** *p* < 0.01 vs. control group.

As shown in Figure 2, compared to the control group, the relative survival rate of MODE-K cells gradually decreased with increasing ZEA concentration when the cells were treated with ZEA for 24 h. The IC_50_ of ZEA on MODE-K cells was 65 μmol/L. Thus, in our study, ZEA with a concentration of 65 μmol/L was added to the MODE-K cells.

### 3.2. Severity of MODE-K Cell Damage by Detection of LDH Activities

**Figure 3:**
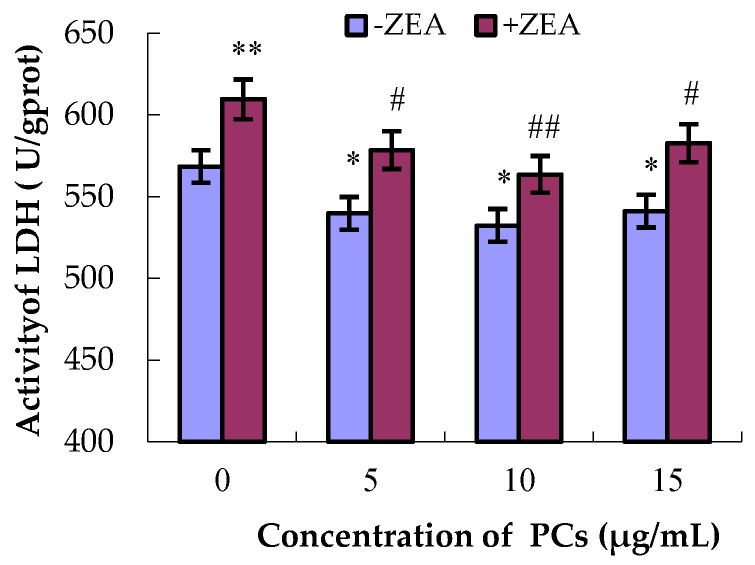
Effects of different concentration of proanthocyanidins (PCs) on the activities of lactate dehydrogenase (LDH) in MODE-K cells with or without 65 μmol/L zearalenone (ZEA). The activities of LDH increased when the cells were exposed to ZEA, while the activities of LDH decreased when the cells were treated with PCs. PCs could also decrease the increase in the activities of LDH when the cells were exposed to ZEA. * p < 0.05 and ** p < 0.01 vs. control group; ^#^ *p* < 0.05 and ^##^ *p* < 0.01 vs. ZEA-treated group.

LDH is a cytoplasmic intracellular enzyme. When cell membranes are damaged, LDH is released into the cell culture medium, and thus the degree of the damage can be measured indirectly by detecting the activity of LDH in the cell culture medium. As shown in Figure 3, compared to the control group, the activities of LDH in the groups PCs5, PCs10, and PCs15 all decreased (*p* < 0.05), whereas the activity of LDH in the ZEA-treated group significantly increased (*p* < 0.01). Compared with the ZEA group, the activities of LDH in the co-treated groups all decreased (*p* < 0.05 and *p* < 0.01). These results show that PCs at concentrations of 5, 10, and 15 μg/mL can alleviate the damaging effects of ZEA on small intestinal epithelial cells.

### 3.3. The Effects of PCs on the Apoptosis of MODE-K Cells Induced by ZEA

**Figure 4:**
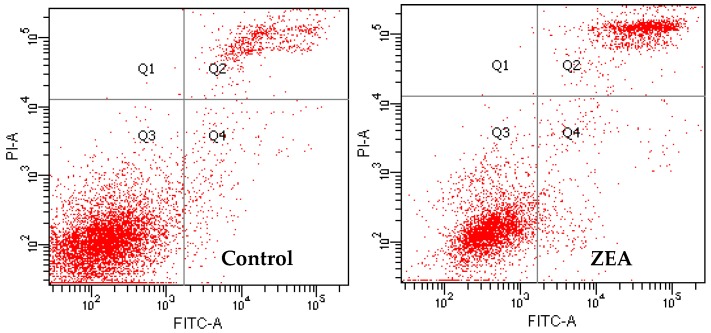

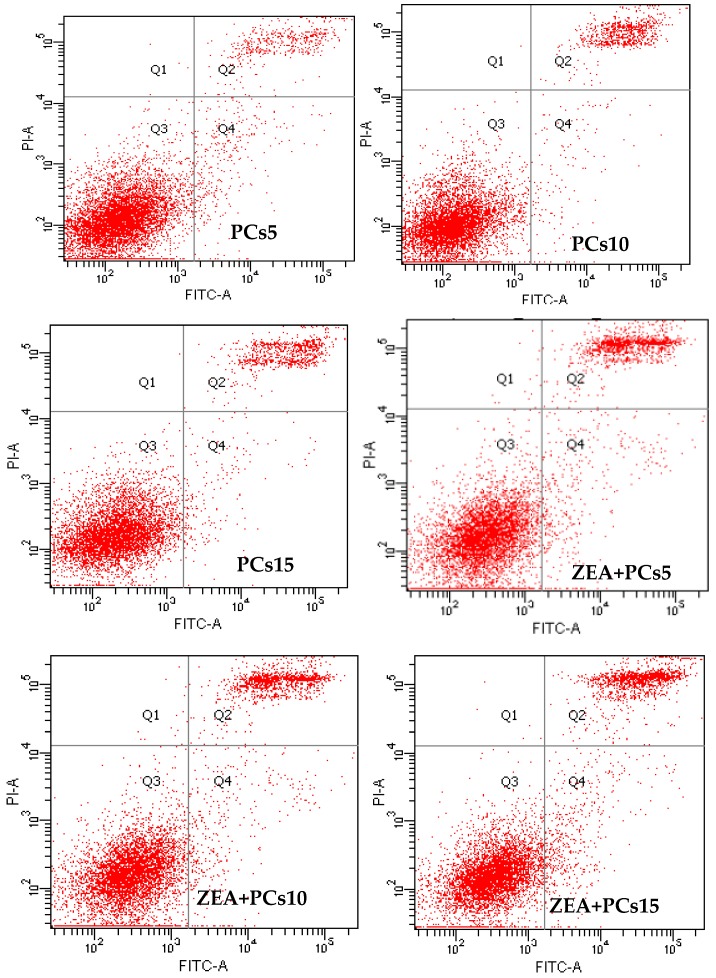
Effect of proanthocyanidins (PCs) on the apoptosis of the MODE-K cells exposed to zearalenone (ZEA).

**Figure 5:**
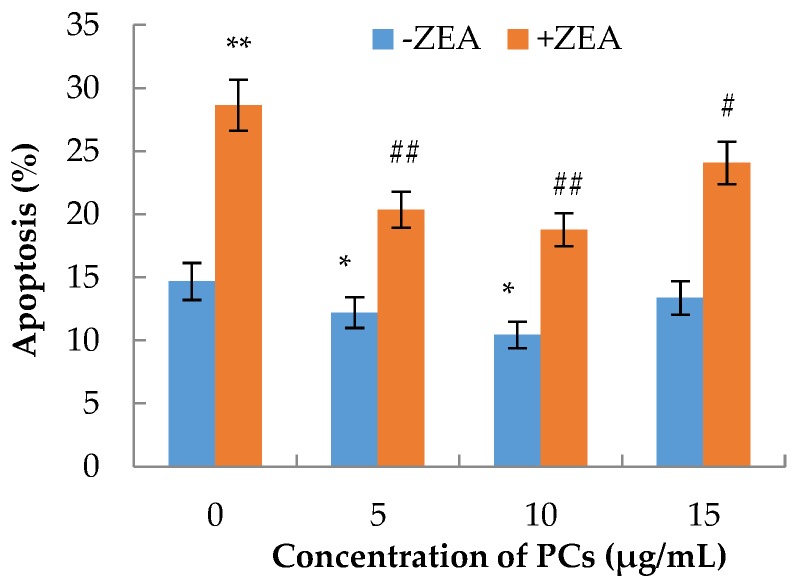
Effects of different concentrations of proanthocyanidins (PCs) on the apoptosis rates of MODE-K cells exposed to zearalenone (ZEA) (65 μmol/L). Rates of apoptosis increased when cells were exposed to ZEA, while the rates of apoptosis decreased when the cells were treated with PCs. The rates of apoptosis also decreased when the cells were co-treated with PCs/ZEA. * *p* < 0.05 and ** *p* < 0.01 vs. control group; ^#^ *p* < 0.05 and ^##^ *p* < 0.01 vs. ZEA-treated group.

The apoptotic rate of each group of cells was calculated by counting the late apoptosis in the Q2 region and early apoptotic cells in the Q4 region. Compared with the control group, the apoptosis rates of MODE-K in the ZEA group significantly increased (*p* < 0.01), whereas the apoptosis decreased when PC concentrations were 5 and 10 μg/mL (*p* < 0.05) (Figure 4 and Figure 5). Compared with the ZEA group, in the co-treated groups, the rate of apoptosis of MODE-K cells significantly decreased (*p* < 0.01) when concentrations of PCs were 5 and 10 μg/mL; furthermore, the rate of apoptosis decreased when the PC concentration was 15 μg/mL (*p* < 0.05).

### 3.4. PCs Suppressed ZEA-Induced Oxidative MODE-K Cell Injury

**Figure 6:**
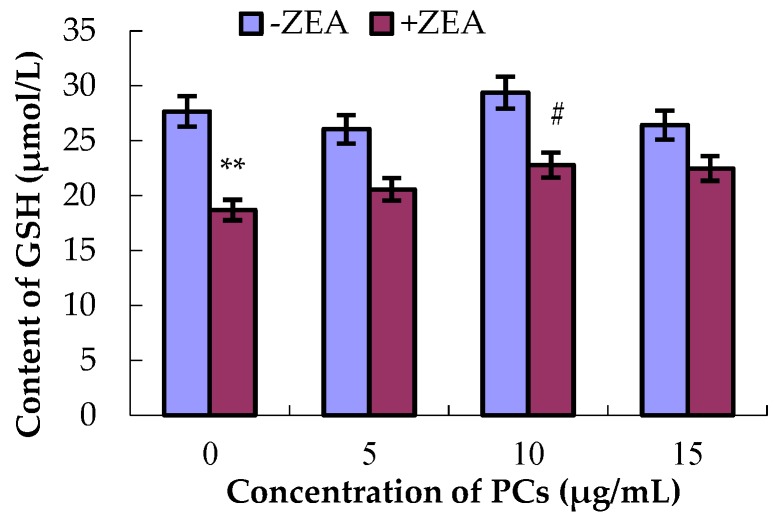
Effects of different concentrations of proanthocyanidins (PCs) on the content of glutathione (GSH) exposed to zearalenone (ZEA) (65 μmol/L). Content of GSH significantly decreased when cells were exposed to ZEA, while the content of GSH did not decrease when cells were treated with PCs. The content of GSH increased when the cells were co-treated with PCs/ZEA. ** *p* < 0.01 vs. control group; ^#^ *p* < 0.05 vs. ZEA-treated group.

**Figure 7:**
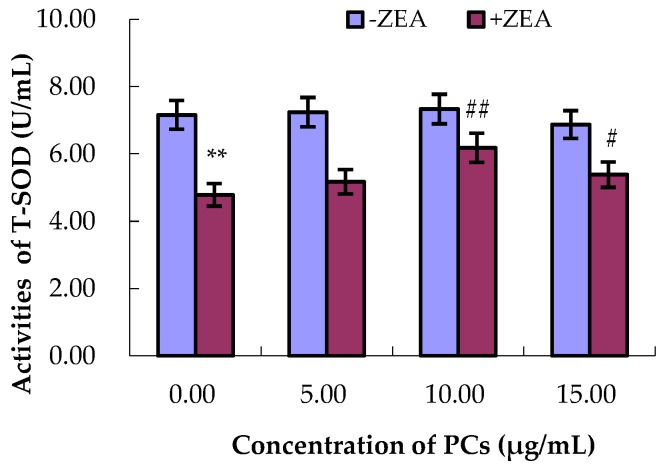
Effects of different concentrations of proanthocyanidins (PCs) on the activities of total superoxide dismutase (T-SOD) exposed to zearalenone (ZEA) (65 μmol/L). The activities of T-SOD decreased when cells were exposed to ZEA, while the activities of T-SOD did not decrease when cells were treated with PCs. The activities of T-SOD increased when the cells were co-treated with PCs/ZEA. ** *p* < 0.01 vs. control group; ^#^ *p* < 0.05 and ^#^^#^ *p* < 0.01 vs. ZEA-treated group.

**Figure 8:**
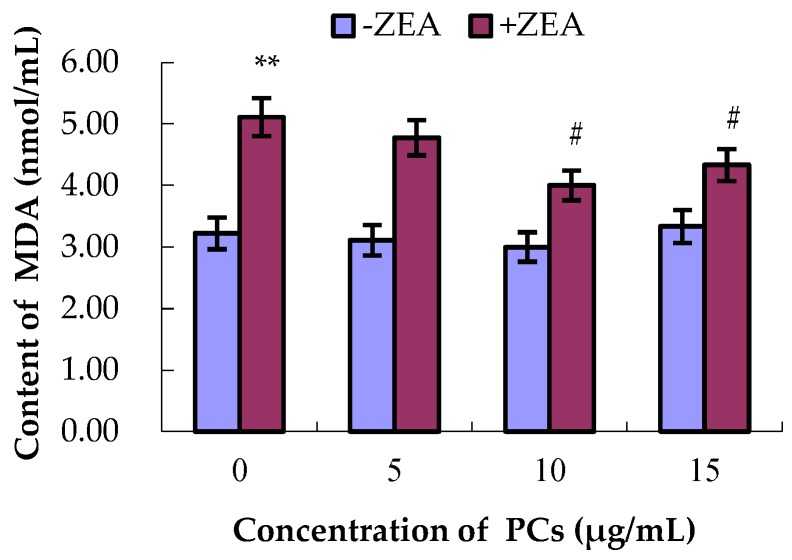
Effects of different concentrations of proanthocyanidins (PCs) on the content of malondialdehyde (MDA) exposed to zearalenone (ZEA) (65 μmol/L). Content of MDA significantly increased when cells were exposed to ZEA, while the content of MDA did not increase when cells were treated with PCs. The content of MDA decreased when the cells were co-treated with PCs/ZEA. ** *p* < 0.01 vs. control group; ^#^ *p* < 0.05 vs. ZEA-treated group.

**Figure 9:**
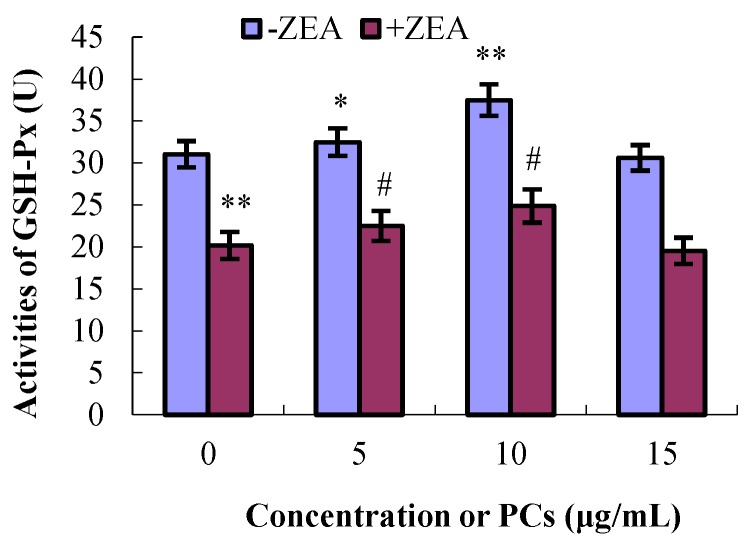
Effects of different concentrations of proanthocyanidins (PCs) on the activities of glutathione peroxidase (GSH-Px) exposed to zearalenone (ZEA) (65 μmol/L). The activities of GSH-Px significantly decreased when cells were exposed to ZEA, while the activities significantly increased when cells were treated with PCs. The activities increased when the cells were co-treated with PCs/ZEA. * *p* < 0.05 and ** *p* < 0.01 vs. control group; ^#^ *p* < 0.05 vs. ZEA-treated group.

As shown in Figure 6, Figure 7, Figure 8 and Figure 9, compared with control group, the content of GSH and MDA and the activities of T-SOD were not significantly difference (*p* > 0.05) for the different concentrations of PCs, whereas the activities of GSH-Px increased when the concentrations of PCs were 5 μg/mL (*p* < 0.05) and 10 μg/mL (*p* < 0.01). Meanwhile, compared with control group, the activities of T-SOD and GSH-Px and the content of GSH significantly decreased (*p* < 0.01) while the content of MDA significantly increased (*p* < 0.01) in the ZEA group. Compared with the ZEA group, the activities of SOD significantly increased in the co-treated groups ZEA/PCs10 (*p* < 0.01) and ZEA/PCs15 (*p* < 0.05), the activities of GSH-Px significantly increased in the co-treated groups ZEA/PCs5 (*p* < 0.05) and ZEA/PCs10 (*p* < 0.05), and the content of GSH significantly increased in the co-treated group ZEA/PCs10 (*p* < 0.05); however, the content of MDA significantly decreased in the co-treated groups ZEA/PCs10 (*p* < 0.05) and ZEA/PCs15 (*p* < 0.05). These results indicated that PCs could suppress ZEA-induced oxidative MODE-K cell injury.

### 3.5. Expression of Bcl-2 and Bax Proteins in MODE-K Cells

**Figure 10:**
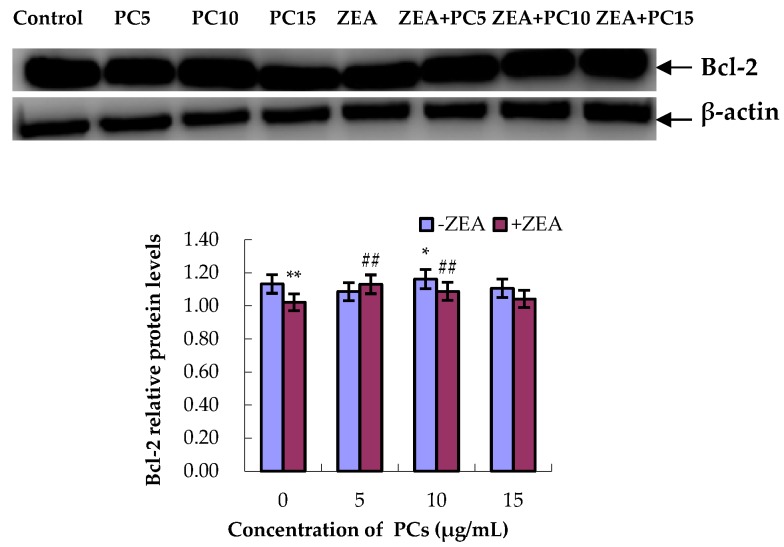
Effects of different concentrations of proanthocyanidins (PCs) on zearalenone (ZEA)-induced relative expression of Bcl-2 protein in MODE-K cells. The relative expression of Bcl-2 protein decreased when cells were exposed to ZEA, while the expression did not decrease when cells were treated with PCs. The expression increased when cells were co-treated with PCs/ZEA. * *p* < 0.05 and ** *p* < 0.01 vs. control group; ^##^ *p* < 0.01 vs. ZEA-treated group.

**Figure 11:**
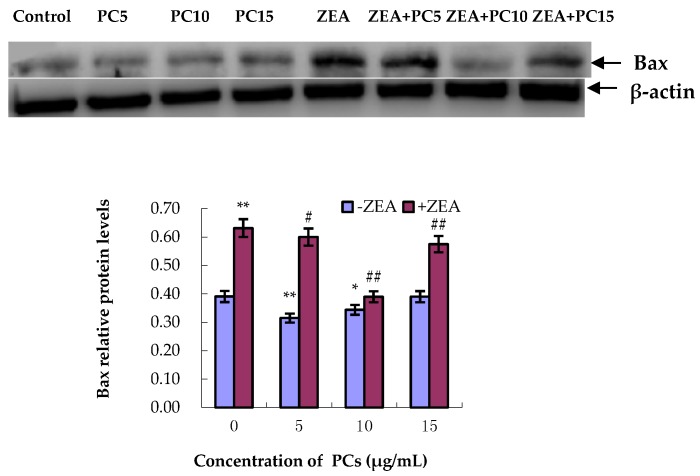
Effects of different concentrations of proanthocyanidins (PCs) on zearalenone (ZEA)-induced relative expression of Bax protein in MODE-K cells. The relative expression of Bax protein increased when cells were exposed to ZEA, while the expression did not increase when cells were treated with PCs. The expression decreased when cells were co-treated with PCs/ZEA. * *p* < 0.05 and ** *p* < 0.01 vs. control group; ^#^ *p* < 0.05 and ^##^ *p* < 0.01 vs. ZEA-treated group.

**Figure 12:**
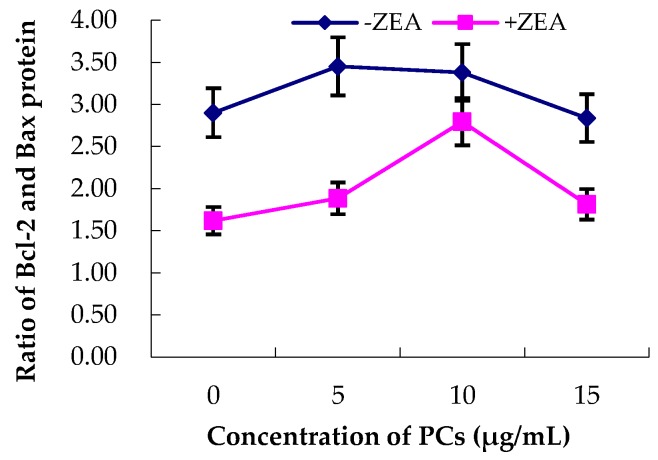
Effects of proanthocyanidins (PCs) on zearalenone (ZEA)-induced ratio of Bcl-2/Bax. The ratio of Bcl-2/Bax was higher when the concentration of PCs was 10 μg/mL in the co-treated PC/ZEA group.

As shown in Figure 10 and Figure 11, compared with the control group, in the ZEA group, the relative expression of the anti-apoptotic Bcl-2 protein significantly decreased (*p* < 0.01), whereas the relative expression of the pro-apoptotic Bax protein significantly increased (*p* < 0.01). However, compared with the ZEA group, there was significant upregulation of the Bcl-2 protein (Figure 10) (*p* < 0.01) in the group co-treated with ZEA/PCs5 and ZEA/PCs10 and downregulation of the Bax protein in all the co-treated groups (Figure 11) (*p* < 0.01). According to the ratio of Bcl-2/Bax relative expression levels (Figure 12), PCs had the highest anti-apoptosis ability at a concentration of around 10 μg/mL.

### 3.6. Expression of the mRNA and Protein Related to ERS-Induced Apoptosis Pathway in MODE-K Cells

**Figure 13:**
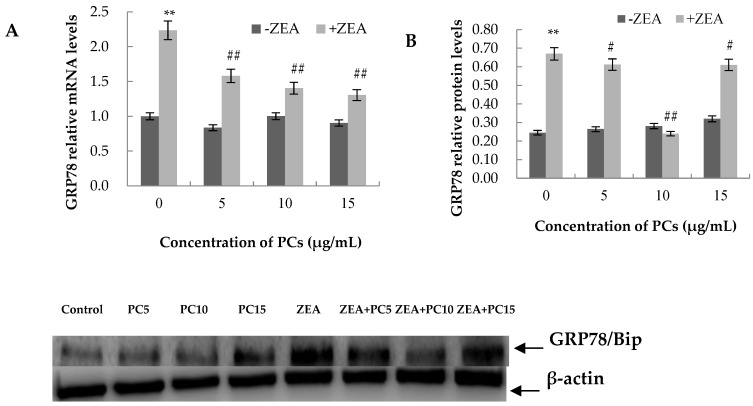
Effects of different concentrations of proanthocyanidins (PCs) on zearalenone (ZEA)-induced relative expression of mRNA (**A**) and GRP78 protein (**B**) in MODE-K cells. PCs could significantly reverse the ZEA-induced increase in GRP78 expression. When the concentration of PCs was 10 μg/mL, PCs could significantly decrease the increase in expressions induced by ZEA. ** *p* < 0.01 vs. control group; *^#^ p* < 0.05 and ^#*#*^ *p* < 0.01 vs. ZEA-treated group.

**Figure 14:**
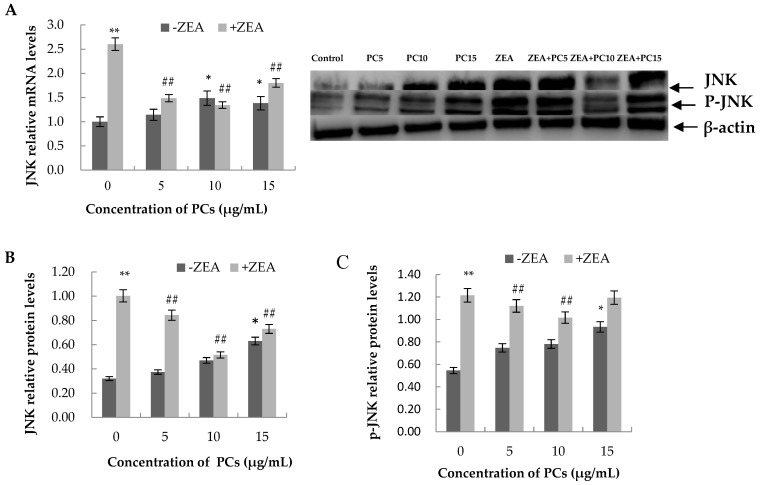
Effects of different concentrations of proanthocyanidins (PCs) on zearalenone (ZEA)-induced relative expression of mRNA and c-Jun N-terminal kinase (JNK) protein (**A** and **B**) in MODE-K cells, and the relative expression of p-JNK protein (**C**). PCs could significantly reverse the ZEA-induced increase in expressions of JNK and p-JNK. When the concentration of PCs was 10 μg/mL, PCs could significantly decrease the increase in protein expressions induced by ZEA. * *p* < 0.05 and ** *p* < 0.01 vs. control group; ^#*#*^ *p* < 0.01 vs. ZEA-treated group.

**Figure 15:**
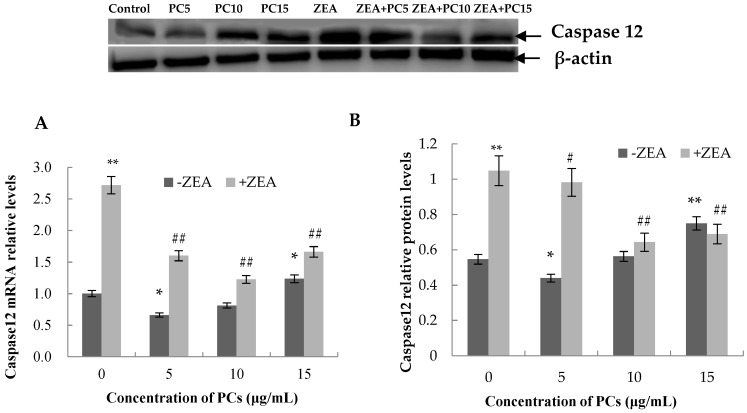
Effects of different concentrations of proanthocyanidins (PCs) on zearalenone (ZEA)-induced relative expression of mRNA (**A**) and cysteinyl aspartate specific proteinase 12 (caspase-12) protein (**B**) in MODE-K cells. When the concentration of PCs was 10 μg/mL, PCs could significantly decrease the increase in expressions induced by ZEA. * *p* < 0.05 and ** *p* < 0.01 vs. control group; *^#^ p* < 0.05 and ^#*#*^ *p* < 0.01 vs. ZEA-treated group.

**Figure 16:**
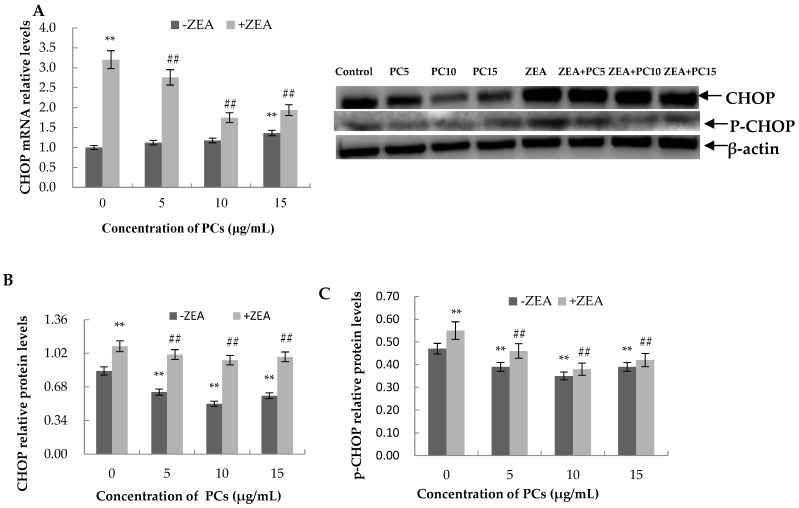
Effects of different concentrations of proanthocyanidins (PCs) on zearalenone (ZEA)-induced relative expression of mRNA and C/EBP homologous protein (CHOP) protein (**A**,**B**) in MODE-K cells and the relative expression of p-CHOP protein (**C**). When the concentration of PCs was 10 μg/mL, PCs could significantly decrease the increase in expressions induced by ZEA. ** *p* < 0.01 vs. control group; ^#*#*^ *p* < 0.01 vs. ZEA-treated group.

As shown in Figure 13, Figure 14, Figure 15 and Figure 16, compared with control group, in the ZEA group, the expressions of mRNA and CHOP, GRP78, JNK, p-JNK, and caspase-12 proteins all increased with significant differences (*p* < 0.01). Compared with the control group, mRNA and protein expressions of GRP78 in all PC groups had no significant differences (*p* > 0.05); the expressions of JNK and p-JNK proteins in the PCs15 group significantly increased (*p* < 0.05), whereas JNK and p-JNK protein expressions did not increase in the PCs5 group (*p* > 0.05). The mRNA and protein expressions of caspase-12 in the PCs5 group decreased (*p* < 0.05), and in the PCs10 group they did not increase (*p* > 0.05), whereas in the PCs15 group they increased (*p* < 0.05). The expressions of CHOP and p-CHOP proteins in all PC/ZEA co-treated groups significantly decreased (*p* < 0.01). As shown in Figure 13, Figure 14, Figure 15 and Figure 16, compared with the ZEA group, the expressions of CHOP, p-CHOP, GRP78, JNK, p-JNK, and caspase-12 proteins all increased with some differences (*p* < 0.05) or with significant differences (*p* < 0.01) in all co-treated PC/ZEA groups. When the PC concentration was 10 μg/mL in the co-treated PC/ZEA group, PCs could significantly reverse the ZEA-induced increase in expressions of mRNA and the protein related to the ERS-induced apoptosis pathway. 

## 4. Discussion

Our results showed that the survival rate of MODE-K cells was inhibited when PCs were used at concentrations of 20 μg/mL or higher, whereas at 10 μg/mL, the growth rate of MODE-K cells increased. The results were consistent with our previous findings that PCs affected the growth of Sertoli TM4 cells [42]. Our results further confirmed that in an appropriate concentration range, PCs had growth-promoting effects, and when the maximum concentration range was exceeded, this could cause cytotoxicity [42,43,44].

The levels of MDA and GSH content and the activities of GSH-Px and SOD are indices of cellular oxidative damage [45]. Our data showed that ZEA induced cytotoxicity in MODE-K cells by reducing cell viability, inhibiting T-SOD and GSH-Px activities and the content of GSH levels, and also increasing the content of MDA. These results further demonstrated that ZEA could induce oxidation, in agreement with other previous studies [10,19,20].

Previous studies have shown that ZEA induces apoptosis in some germ cells, such as murine ovarian germ cells [46], mouse endometrial stromal cells [47], and Sertoli cells [42,48,49]. ZEA can also induce apoptosis in porcine intestinal epithelial cells (IPEC-J2 cells) [50] and human large intestine cells (HCT116) [10]. In this study, we confirmed that ZEA causes apoptosis in mouse intestinal epithelial cells (MODE-K cells). As demonstrated in the previous studies, oxidative stress can induce apoptosis of the cells [51,52,53], and combined with the data in this study, we determined that ZEA induces apoptosis in MODE-K cells via oxidative stress. Intestinal epithelial cells are the first line of defense against the invasion of foreign poisons. We speculate that ZEA could damage the tight-junction barrier of intestinal epithelial cells as it may cause intestinal epithelial cell apoptosis. However, our presumption would need further investigation in order to determine whether ZEA has an effect on the expression of tight-junction-related genes and proteins in intestinal epithelial cells.

Few reports describe the mechanism of reduced apoptosis induced by ZEA. As ZEA can induce apoptosis by causing oxidative stress and because PCs have a strong antioxidant capability, we aimed to determine whether PCs can prevent intestinal epithelial cell apoptosis induced by ZEA. Our data showed that PCs, with their strong antioxidant capability, corrected a decrease in the antioxidant enzyme activities of T-SOD and GSH-Px and in the content of GSH. They also reduced an increase in the content of MDA caused by ZEA, decreased the number of apoptosis MODE-K cells, and revised the expressions of the apoptosis gene Bax and anti-apoptosis gene Bcl-2. According to our results, we conclude that PCs can reduce oxidative stress and then ameliorate ZEA-induced apoptosis of intestinal epithelial cells.

ERS can make three kinds of transmembrane signal proteins (IRE1, PERK, and GRP78) dissociate from the chaperone protein GRP78 on the endoplasmic reticulum membrane. These activated receptor proteins can activate the unfolded protein response (UPR) to protect the cells; however, long-term ERS will lead to the activation of the apoptotic proteins CHOP, JNK, and caspase-12 and promote apoptosis of the cells [54,55,56,57]. Our results showed ZEA can induce ERS, as it promoted the expression of mRNA and protein of the ER resident chaperone GRP78, the most important ERS marker [18]. PCs can also effectively alleviate ERS because they revise the elevation expression of the GRP78 protein induced by ZEA. Our results also found that although PCs could reduce the normal mild ERS of the cells at low concentration levels (5 and 10 μg/mL), they also induced ERS at high concentrations, as the results showed that when the concentration of PCs was 15 μg/mL, the expressions of caspase-12, JNK, p-JNK, and Bax had rising trends. Additionally, at this concentration, PCs did not significantly reduce the increase in the expression of these proteins induced by ZEA. Previous studies have also shown that PCs at a high dose (500 mg/mL) could increase cell death [43] and that procyanidin dimmer B2 (50 μM) was more cytotoxic than cyclophosphamide in MCF-7 human breast adenocarcinoma cells [44]. However, it was generally safe when the oral intake of PCs was up to 2500 mg for 4 weeks in humans [58], and a rat diet containing 2% of PCs produced no observed adverse effect level (NOAEL) [59]. Moreover, excessive addition of any substance to cells can cause cell damage. Thus, combined with our results, we believed that in vitro, high doses of PCs may induce toxicity, while at low doses, no toxicity is observed, which could protect against the oxidative stress injury to cells.

The expression of the CHOP protein increases when ERS occurs [55], thereby inhibiting the expression of Bcl-2 cells and promoting cell apoptosis [60]. The JNK protein is mainly cytoplasmic. JNK converts to its phosphorylated form p-JNK and enters the nucleus to exert its activity when cells are stimulated, resulting in nuclear activation of the transcription factor (c-Jun) [61] and finally activating Bax and other pro-apoptotic proteins [54]. Caspase-12 exists in the form of a caspase enzyme (procaspase-12) that is not bioactive when the cells are in a normal state. Procaspase-12 is converted to caspase-12, which can activate the downstream caspase-3 and caspase-9 to then induce apoptosis when cells are stimulated by external negative factors [62]. Our results showed that ZEA could significantly induce the mRNA and protein expression of CHOP, JNK, and caspase-12, which are the protein markers of the three signal pathways of PERK/ATF6 and IRE1/caspase-12, related to ERS-induced apoptosis [55,56,57]. Previous studies have suggested that ZEA induces apoptosis and death via an ERS-dependent signaling pathway [10,53,63]. Our results indicated that ZEA induced apoptosis in MODE-K cells via ERS mediated through the PERK, IRE1, ATF6, and caspase pathways. The addition of a suitable concentration of PCs can significantly reduce the expression of these specific genes and proteins. These results indicated that a certain concentration of PCs can relieve the effect of ZEA on ERS. Combined with our results, that PCs reduced the protein expression of the related apoptosis proteins Bcl-2 and Bax induced by ZEA, we confirmed that PCs could protect the small intestinal epithelial cells of mice from ZEA-induced apoptosis via inhibition of ERS-induced apoptosis pathways.

## 5. Conclusions

In conclusion, in this study, we further proved that ZEA induces apoptosis via an ERS-dependent signaling pathway and that PCs within a certain range could attenuate ZEA-induced apoptosis in MODE-K cells via inhibition of the ERS-induced apoptosis pathway.

## Figures and Tables

**Table 1:** Primers.

Gene	Accession No.	Primer Sequence (5′–3′)	Product Length
*β-actin*	BC138614.1	Forward: CTGTCCCTGTATGCCTCTG	221 bp
		Reverse: TTGATGTCACGCACGATT	
*Caspase-12*	NM_009808.4	Forward: CTCAATAGTGGGCATCTGGGT	151 bp
		Reverse: GAAGGTAGGCAAGACTGGTTC	
*CHOP*	NM_001290183.1	Forward: TTCTCCTTCATGCGTTGCTTC	218 bp
		Reverse: AAAACCTTCACTACTCTTGACCCTG	
*JNK*	NM_001310452.1	Forward: TCCTCCAAATCCATTACCTCC	149 bp
		Reverse: CTCCAGCACCCATACATCAAC	
*GRP78*	NM_001163434.1	Forward: CGCTGGGCATCATTGAAGTAA	145 bp
		Reverse: GAGGTGGGCAAACCAAGACAT	
